# Persistent primary hyperparathyroidism caused by an ectopic adenoma in the piriform sinus: case report and review of the literature

**DOI:** 10.3389/fonc.2024.1431561

**Published:** 2024-08-19

**Authors:** Chiara Sardella, Veronica Seccia, Massimo Giambalvo, Laura Pierotti, Elisa Dinoi, Simone Della Valentina, Elena Pardi, Gabriele Materazzi, Iacopo Dallan, Stefano Berrettini, Filomena Cetani

**Affiliations:** ^1^ Endocrine Unit, University Hospital of Pisa, Pisa, Italy; ^2^ Otolaryngology, Audiology and Phoniatric Operative Unit, University Hospital of Pisa, Pisa, Italy; ^3^ Department of Clinical and Experimental Medicine, University of Pisa, Pisa, Italy; ^4^ Skull Base and Rhino-Orbital Surgery Unit, University Hospital of Pisa, Pisa, Italy

**Keywords:** ectopic parathyroid adenoma, parathyroid glands, transoral robotic parathyroidectomy, pyriform sinus, persistent primary hyperparathyroidism

## Abstract

**Introduction:**

Primary hyperparathyroidism (PHPT) is a common endocrine disorder in which surgery is the only curative therapy. Ectopic parathyroid adenoma in the pyriform sinus resulting from a pathological migration of parathyroid glands along the embryological development is a rare cause of PHPT. We describe a case of a persistent primary hyperparathyroidism after previous unsuccessful surgery due to an ectopic parathyroid adenoma within the pyriform sinus and we review the previous reports on this issue.

**Case presentation:**

A 62-year-old woman was referred for persistent hypercalcemia following unsuccessful cervical exploratory surgery. Cervical ultrasound did not detect any parathyroid abnormalities. At variance, ^99m^Tc-sestamibi SPECT/CT and CT scan of the neck identified a parathyroid adenoma in the left pyriform sinus, which was confirmed by endoscopy. The patient was successfully treated by transoral robotic resection and the pathology confirmed a parathyroid adenoma.

**Conclusions:**

The ectopic parathyroid adenoma in the pyriform sinus is so uncommon that only fourteen cases have been reported. However, the pyriform sinus should be considered a possible location of ectopic parathyroid glands, especially in the setting of persistent or recurrent PHPT after parathyroid surgery.

## Introduction

Primary hyperparathyroidism (PHPT) is a common endocrine disease caused by a single parathyroid adenoma in 90% of cases (1). Parathyroid surgery is the only option for curing PHPT and, in experienced hands it is a safe and well-tolerated operation with success rates of 95-97% (1–3). However, persistent or recurrent PHPT still occurs in 2.5-10% of the cases (1–6).

Persistent PHPT is defined as hypercalcemia within 6 months after surgery whereas recurrent PHPT is defined as a new finding of hypercalcemia after a period of 6 months in patients successfully operated and in whom normocalcemia was previously documented (3). Reoperation for persistent or recurrent PHPT compared with the initial surgery, are associated with higher complications rates (4–6). Therefore, in patients requiring a surgical approach for persistent or recurrent PHPT, preoperative localization imaging studies (i.e., 18F-flurocholine PET/CT, and 4D-CT) are mandatory in order to detect multiglandular disease and/or parathyroid ectopic tissue (3).

Ectopic parathyroid glands arise from an aberrant migration of parathyroid glands along the embryological development and ectopic parathyroid adenomas can frequently lead to persistent post-surgical PHPT (7–12). The prevalence of ectopic parathyroid glands ranges from 2% to 43% in anatomical series and up to 16% and 14% in patients with PHPT and secondary hyperparathyroidism, respectively (6–8). Ectopic inferior parathyroids are most frequently located in the anterior mediastinum, often in association with the thymus or the thyroid gland, while ectopic superior parathyroids are commonly found in the tracheoesophageal groove and retroesophageal region (9–12).

We report a patient with persistent PHPT following surgery wherein an ectopic parathyroid adenoma was identified within the lefty pyriform sinus. Additionally, we provide a review of previous reports concerning ectopic parathyroid glands discovered in the pyriform sinus. This case adds to the limited body of literature on this rare anatomical variant and highlights the challenges associated with its diagnosis and management.

## Case presentation

The patient, a 62-yr-old woman, was referred to our outpatient clinic for further evaluation of primary hyperparathyroidism (PHPT) persistent after surgery. Her past medical history was notable for hypercalcemia detected 2 years earlier during routine blood examination. Further exams confirmed elevated serum concentrations of calcium (13.8 mg/dl, normal range, 8.1-10.4) and intact PTH levels (54 pg/ml; normal range, 6-36). Neck ultrasound showed a 0.9-cm hypoechogenic lesion posterior to the upper portion of the left thyroid lobe consistent with an enlarged parathyroid gland. Planar ^99^Tc-sestamibi scan displayed an increased uptake at the same site. She underwent surgery. At neck exploration no apparent enlarged parathyroid tissue was evident at the left side of the neck or in any other eutopic or ectopic location in the neck. Additionally, a 7-mm lesion at the left side of the thyroid lobe and an 8-mm nodule at the right side of the neck were removed. However, the nature of these lesions was described as unclear. Despite the surgical procedure, there was no decline of intraoperative PTH (baseline 83 vs 85 pg/ml at the close of the surgery) predicting un unsuccessful surgery. No pathological parathyroid tissue was seen on histology. Histopathology showed two lymph nodes with hyperplasia. After surgery, serum calcium and PTH levels remained elevated (11.1 mg/dl and 47 pg/ml, respectively).

**Figure 1:**
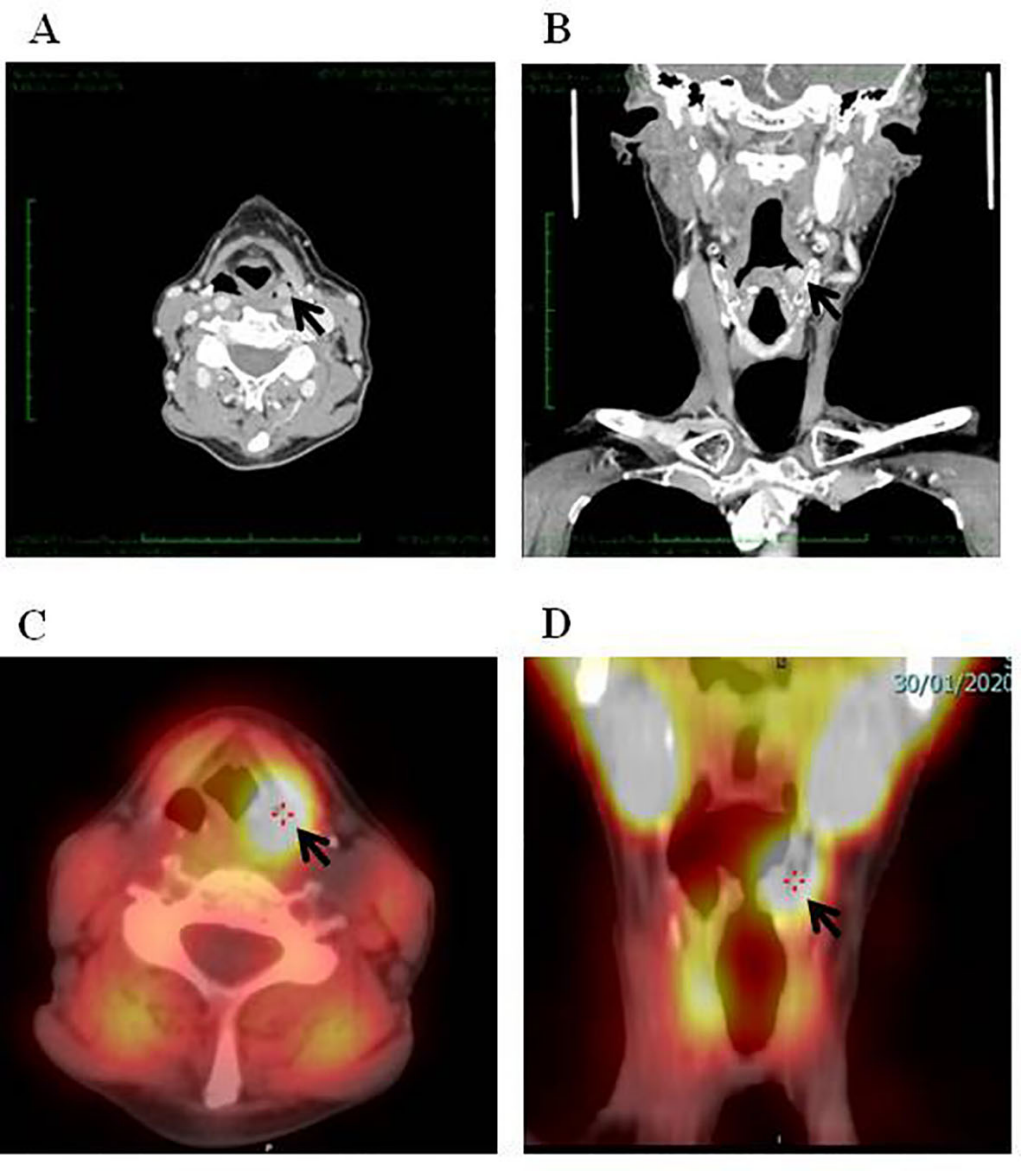
Parathyroid adenoma of the left pyriform identified by neck CT (**A**, coronal; **B**, axial) and ^99m^TC-MIBI SPECT/CT (**C**, coronal; **D**, axial).

**Figure 2:**
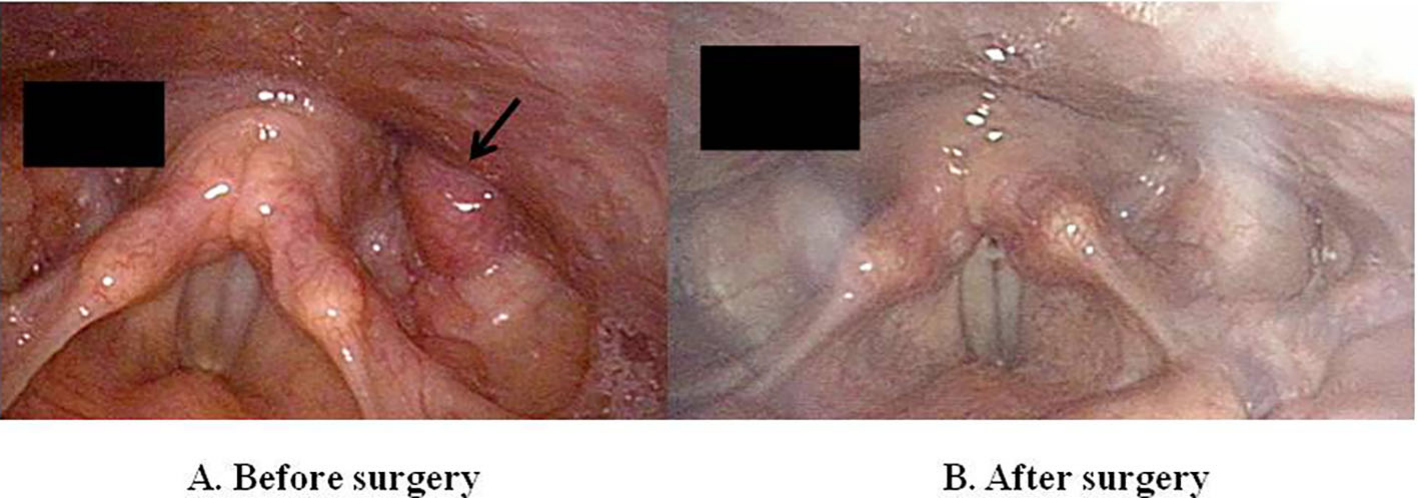
Pre-operative laryngoscopy image revealing a mass in the left pyriform sinus (arrow) **(A)**. Post-operative laryngoscopy image showing successful excision of parathyroid adenoma after trans-oral robotic parathyroidectomy **(B)**.

In December 2021, the patient was referred to our outpatient clinic for further evaluation. The past medical history was unremarkable. Family history was negative for PHPT or other endocrine tumors. The patient was in good health. Physical examination was negative, the surgical scar of her neck was healing well. Laboratory investigations confirmed mild hypercalcemia and mildly elevated PTH levels (**Table 1**). Twenty-four-hour urinary calcium were slightly elevated and bone turnover markers were normal (**Table 1**). Under supplementation of 8750 UI per week of cholecalciferol, 25-hydroxy vitamin D [25(OH)D_3_] was 31.5 ng/mL. Renal ultrasound did not show a bilateral nephrolithiasis and/or nephrocalcinosis. Bone mineral density (BMD) measured by dual-energy X-ray absorptiometry (DXA) showed a reduction in bone mass at lumbar spine, femur and distal 1/3 radius. There were no signs of vertebral fracture at vertebral fracture assessment (VFA). The neck ultrasound did not show enlarged parathyroid glands. However, the contrast-enhanced neck CT identified a 7-mm nodular lesion within the left pyriform sinus (**Figures 1A, B**). Subsequent imaging with single-photon emission CT (SPECT-CT) revealed increased radiotracer uptake in this same area suggestive of an ectopic left pyriform sinus parathyroid adenoma (**Figures 1C, D**). Fiber optic laryngoscopy showed an extrinsic 1-cm mass pushing against the posterior wall of the left pyriform sinus (**Figure 2A**). Patient was recommended to undergo trans-oral robotic parathyroidectomy (PTX), which was performed in November 2022 The FK retractor was employed for visualization of the mass situated in the left pyriform sinus. Utilizing the da Vinci SI robotic system outfitted with a 5-mm Maryland Dissector and a 5-mm flat-tipped monopolar cautery instrument, a hypopharyngeal incision measuring 1 cm was created utilizing electrocautery, followed by the careful dissection of the mass from the adjacent tissues employing a combination of blunt dissection and electrocautery techniques (**Figure 2B**). Baseline PTH was 158 pg/ml, which dropped to 110 pg/ml within 10 minutes post-surgery. The pathology report revealed a parathyroid chief cell adenoma measuring 12 x 5 x 3 mm.

**Table 1:** Biochemical and DXA parameters before and after surgery.

Parameters	Before surgery	12 months after surgery	Reference range
Total calcium (mg/dL)	11.4	9	8.6-10.2
Ionized calcium (mmol/L)	1.55	1.2	1.13-1.32
Phosphate (mg/dL)	2.2		2.5-4.5
Magnesium (mg/dL)	2.2		1.7-2.5
Creatinine (mg/dL)	0.58	0.66	0.7-1.2
eGFR	101	99	
PTH (pg/mL)	72	30	8-40
25(OH)D_3_ (ng/mL)	31.5	35.3	20-100
BSAP (mcg/L)	25	9	4.7-27.1
Osteocalcin (mcg/L)	25	7.1	6.8-34
S-CTX (mcg/L)	0.453	0.443	0.034-1.037
24-h urinary calcium (mg)	389	140	100-320
Lumbar spine BMD (g/cm2)	0.661	0.725	
Lumbar spine T-score	-3.5	-2.9	
Femoral neck BMD (g/cm2)	0.606	0.608	
Femoral neck T-score	-2	-2.2	
Total hip BMD (g/cm2)	0.747	0.703	
Total hip T-score	-1.6	-2	
1/3 distal radius BMD (g/cm2)	0.497	0.470	
1/3 distal radius T-score	-3.6	-3.7	

eGFR, estimated glomerular filtration rate; 25(OH)D, 25-hydroxy vitamin D; BSAP, bone specific alkaline phosphatase; S-CTX, serum carboxy-terminal collagen crosslinks; BMD, bone mineral density.

One month after surgery serum concentrations of calcium and PTH were 9.4 mg/dl and 26 pg/ml, respectively, indicating a remission of PHPT. Twelve months after surgery serum calcium and PTH were normal (**Table 1**). Serum carboxy-terminal collagen crosslinks (S-CTX) values were normal. After surgery, DXA showed approximately a 9% increase in BMD at lumbar spine, while BMD values at the femur and 1/3 distal radius remained stable (**Table 1**).

## Systematic review of the literature

We conducted a review of the literature of cases of parathyroid glands within the pyriform sinus. We performed a systematic search in Pubmed from 1992 to February 2024. We used the following key words: “ectopic parathyroid glands”, “pyriform sinus”, “ectopic parathyroid adenoma”, “persistent hyperparathyroidism”, “and ectopic parathyroid tissue”. We retrieved all eligible studies and we checked in their references in order to finding additional relevant articles.

The search retrieved a total of 20 references. Twelve studies were eligible and included in the systematic review (**Table 2**).

**Table 2:** Case reports detailing ectopic parathyroid adenoma/hyperplasia within the pyriform sinus.

Authors	Year of Publication	N. of case reported	Age at diagnosis/Sex	Clinical presentation	Sinus site	Histology	Cured (Yes/Not)
Stojadinovic A et al (13).	1998	1	68 yrs/female	Symptomatic PHPT	Left	Parathyroid adenoma	Yes
Fukumoto A et al. (14)	2002	1	38 yrs/female	Hyperparathyroidism secondary to renal failure Symptom caused by mass effect	Right	Parathyroid hyperplasia	Not
Dedivitiis RA et al. (15)	2004	1	65 yrs/female	Symptomatic PHPT with renal complications	Left	Parathyroid adenoma	Yes
Murakami N et al. (16)	2012	1	46 yrs/female	Persistent asymptomatic PHPT in patient with multiple endocrine neoplasia type 1	Right	Parathyroid hyperplasia	Yes
Guevara N et al. (17)	2013	1	44 yrs/female	Persistent asymptomatic PHPT	Left	Parathyroid adenoma	Yes
Smith MM et al. (18)	2016	1	55 yrs/female	Symptomatic PHPT	Left	Parathyroid adenoma	Yes
Kim J BS et al. (19)	2017	3	68 yrs/female	Symptomatic PHPT with renal complications	Right	Parathyroid adenoma	Yes
			58 yrs/female	Persistent symptomatic PHPT with renal and bone complications	Right	Parathyroid adenoma	Yes
			66 yrs/female	Persistent symptomatic PHPT Symptom caused by mass effect	Right	Parathyroid adenoma	Yes
Mulleman T et al. (20)	2018	1	Middle aged/female	Hyperparathyroidism secondary to renal failure Symptom caused by mass effect	Bilateral	Parathyroid adenomas	Not
Connolly MJ (21)	2019	1	53 yrs/female	Asymptomatic PHPT	Right	Parathyroid adenoma	Yes
Hsieh MP et al. (22)	2020	1	70 yrs/female	Persistent PHPT	Right	Parathyroid adenoma	Yes
Zenno A et al. (23)	2023	1	9 yrs/female	Persistent symptomatic PHPT	Left	Parathyroid adenoma	Yes
Sina EM et al. (24)	2023	1	41 yrs/female	Asymptomatic PHPT	Left	Parathyroid adenoma	Yes

A total of 14 cases of ectopic parathyroid adenomas in the pyriform sinus have been reported in the literature from 1998 to 2023 (**Table 2**). The patients were all females with age range from 9 to 70 years-old. Ectopic parathyroid tissue within the pyriform sinus was the cause of PHPT in 12 (86%) cases and in two cases (14%), the ectopic parathyroid tissue was associated with hyperparathyroidism secondary to renal failure. Parathyroid adenomas were most commonly detected in the right pyriform sinus accounting for 50% of cases and in only one patient had presented parathyroid ectopic tissue identified bilaterally within the pyriform sinus.

The surgical removal of the parathyroid adenoma was curative for patients with PHPT but not for those affected by secondary hyperparathyroidism to renal failure (**Table 2**).

## Discussion

Successful surgery for PHPT is dependent on the experience of the operating surgeon, the presence of multiglandular disease or parathyroid carcinoma and the anatomic location of the parathyroid glands. Indeed, the presence of ectopic parathyroid glands can be a diagnostic challenge for clinicians and lead to persistent PHPT (1–3).

Ectopic locations of the parathyroid glands are related to an aberrant migration of parathyroid glands along the embryological development. Indeed, the superior parathyroid glands arise from the fourth pharyngeal pouch and typically descend along with the lateral lobes of the thyroid gland. They commonly reside in the vicinity of the cricothyroid junction and the dorsum of the upper pole of the thyroid. However, occasionally, they can be found in more unusual locations such as the retropharyngeal or retroesophageal space. On the other hand, the inferior parathyroid glands arise from the third pharyngeal pouch and descend with the thymus gland toward the mediastinum. Consequently, they are usually located in the lower pole of the thyroid gland or within the thymic tongue. However, they can also be found in other ectopic locations, including the upper neck, the lateral neck or even within the mediastinum (9–12).

The prevalence of ectopic parathyroid glands ranges from 9% to 22% in different studies. These glands can contribute to persistent or recurrent PHPT in up to 8% of cases of (7–12). Ectopic parathyroid adenomas are primarily located in specific regions such as tracheoesophageal groove, within the thymus or less frequently within the thyroid gland itself (10, 12). Interestingly, ectopic parathyroid adenomas often exhibit distinct characteristics compared to orthotopic (normally located) parathyroid adenomas. They tend to have higher serum calcium levels and larger tumor sizes. Specifically, serum calcium levels in cases of ectopic adenomas have been reported to be significantly higher (12.6 mg/dl compared to 11.4 mg/dl) and tumor sizes larger (2.5 cm compared to 1.9 cm) than those of orthotopic adenomas (10).

The patient presented herein, despite previous bilateral neck exploration, had a persistent PHPT due to an ectopic parathyroid gland within the pyriform sinus. At the time the patient was examined at our hospital, she had an asymptomatic PHPT with moderate hypercalcemia and osteoporosis at lumbar spine and at the one-third distal radius indicating the need for remedial surgery to prevent potential complications associated with long-term untreated PHPT. Imaging studies identified a 7-mm lesion within the left pyriform sinus providing important localization information for surgical planning. Endoscopic exploration directly visualized the ectopic parathyroid gland within the left pyriform sinus, which was histologically confirmed as parathyroid adenoma.

The pyriform sinus is a rare ectopic location for parathyroid glands. The proposed mechanism for ectopic parathyroid glands in this location involves a developmental anomaly where the superior parathyroid gland primordia fail to lose connection with the pharyngeal wall during embryonic development (25). This results in a parathyroid gland with a submucosal location internal to the plane of the inferior constrictor muscle, as observed in this case.

The first two cases of ectopic parathyroid glands located within the pyriform sinus were reported in 1982 and involved ectopic parathyroid glands in the pyrform sinus that were accidentally removed during surgery for laryngopharyngeal cancer (25). Subsequently, a total of fourteen cases of parathyroid adenomas located within the pyriform sinus have been described in the literature (13–24). In 2023, Zenno et al. reported a case involving a 9-year-old girl with a persistent PHPT following bilateral neck exploration (23). This case underscores the ongoing challenges associated with the diagnosis and management of ectopic parathyroid glands, particularly in young patients.

Among the reported cases, ectopic adenomas within the pyriform sinus were found in patients with various underlying conditions, including 6 patients with PHPT (13, 15, 18, 19, 21, 24), 6 with persistent or recurrent PHPT (16, 17, 19, 22, 23) and 2 with secondary hyperparathyroidism due to renal failure (14, 20). From a clinical perspective, PHPT presented disease-related symptoms in 7 cases (13, 15, 18, 19, 23) and, complications in 3 patients (15, 19). Additionally, 3 subjects reported symptoms attributable to mass effect such as dysphagia or sensation of a lump in the throat (14, 19, 20) (**Table 2**). Histological examination of excised tissue revealed different findings among the patients. While the majority of the patients had parathyroid adenomas (13, 15, 17–24), two cases showed parathyroid hyperplasia (14, 16), notably, one patient with multiple endocrine neoplasia type 1 (16) and one with secondary hyperparathyroidism due to renal insufficiency (14) (**Table 2**). These findings underscore the diverse clinical presentations and histopathological characteristics associated with ectopic parathyroid glands in the pyriform sinus.

Preoperative localization studies are essential components of the preoperative work-up of patients with persistent or recurrent PHPT. These studies play a crucial role in identifying the location of abnormal parathyroid glands, including ectopic tissue, and determining the extent of the disease (3).

In our case, conventional imaging (neck ultrasound and planar ^99^Tc-sestamibi parathyroid scan) before the first surgery was inconclusive whereas neck CT and ^99m^Tc-sestamibi SPECT/TC performed before the reoperation identified the parathyroid lesion within the left pyriform sinus. Indeed, SPECT images coupled to a simultaneously acquired CT are superior to planar studies for anatomical localization of parathyroid tissue especially in case of ectopic glands and altered neck anatomy (26). Moreover, a second-line imaging techniques such as 4D-CT or F18-choline (FCH) PET-CT is required in patients with persistent or recurrent hyperparathyroidism after surgery. In a prospective study of 45 patients who underwent parathyroid reoperation, 4D-CT displayed a higher sensitivity compared to ^99m^Tc-sestamibi SPECT/TC and neck US (88%, 54% and 21%, respectively) (26). Similarly, it has been reported that FCH PET/CT demonstrated a sensitivity comparable to that of 4DCT (84%) in patients with persistent primary hyperparathyroidism (PHPT) following unsuccessful prior surgery (27). Of note, laryngoscpy has been considered as a localization procedure in patients with suspected parathyroid adenoma in the pyriform sinus (13–24).

From a therapeutic point of view, surgery is the mainstay of the treatment of the ectopic parathyroid adenomas. Conventional open surgical techniques have traditionally been the standard approach for parathyroid adenoma excision (15, 20, 21, 23, 24). However, over the past 15–20 years, there has been a notable shift towards adopting minimally invasive methods, including transoral endoscopic approaches, aimed at minimizing visible scarring for patients (13, 14, 16, 17, 19, 22).

Notably, the utilization of transoral CO2 laser surgery was initially documented by Stojadinovic et al. in 1998 (13). Subsequently, in 2013, Guevara et al. (17) reported the second documented case of employing an endoscopic CO2 laser-assisted technique for resecting an ectopic parathyroid adenoma within the pyriform sinus. This technique was later applied in three additional patients (19, 22).

Since the introduction of the da Vinci robotic system in head and neck surgery in 2005, numerous transoral robotic surgical procedures have been elucidated (28). In December 2009, the FDA granted approval for robotic surgery in select cases of oropharyngeal and laryngeal cancers, as well as in multi-level sleep apnea surgeries. Robotic techniques have gained popularity among patients due to their purported benefits, including expedited recovery, enhanced cosmetic outcomes, and reduced postoperative pain. The integration of surgical robotics offers superior visualization, extended range of motion, and enhanced precision, potentially leading to improved surgical outcomes compared to previously described transoral techniques. It is worth noting that Transoral Robotic Surgery (TORS) has been reported in other two cases similar to ours, as documented by Smith et al. in 2016 (18) and by Kim et al. in 2017 (19).

In conclusion, ectopic parathyroid adenomas within the pyriform sinus are exceedingly rare. Their atypical location poses a challenge for detection during standard parathyroid workup. Due to their rarity and unusual location, ectopic parathyroid adenomas within the pyriform sinus are likely to be missed on routine preoperative localization studies, including neck ultrasound and planar 99mTc-sestamibi scan. However, clinicians should consider the pyriform sinus as a potential location of ectopic parathyroid glands, particularly in cases of persistent or recurrent PHPT following parathyroid surgery. When standard preoperative localization studies fail to detect abnormal parathyroid glands or when there is suspicion of ectopic location, advanced imaging modalities such as neck CT and 99mTc-sestamibi SPECT/CT, as well as second-line techniques like 4D-CT or F18-choline PET-CT, should be utilized to improve sensitivity and accuracy in detecting parathyroid lesions. Awareness of the possibility of ectopic parathyroid glands within the pyriform sinus is crucial for optimizing patient care and surgical outcomes in cases of persistent or recurrent PHPT. Consideration of this rare anatomical location may help prevent delays in diagnosis and facilitate successful surgical intervention.

## Data Availability

The raw data supporting the conclusions of this article will be made available by the authors, without undue reservation.

## References

[1] Bilezikian JP Khan AA Silverberg SJ Fuleihan GE Marcocci C Minisola S . Evaluation and management of primary hyperparathyroidism: summary statement and guidelines from the fifth international workshop. J Bone Miner Res. (2022) 37:2293–314. doi: 10.1002/jbmr.4677 36245251

[2] Bilezikian JP Silverberg SJ Bandeira F Cetani F Chandran M Cusano NE . Management of primary hyperparathyroidism. J Bone Miner Res. (2022) 37:2391–403. doi: 10.1002/jbmr.4682 36054638

[3] Bollerslev J Rejnmark L Zahn A Heck A Appelman-Dijkstra NM Cardoso L . European expert consensus on practical management of specific aspects of parathyroid disorders in adults and in pregnancy: recommendations of the ESE educational program of parathyroid disorders. Eur J Endocrinol. (2021) 186:33–63. doi: 10.1530/EJE-21-1044 PMC8789028 34863037

[4] Wells SA Jr Debenedetti MK Do-herty GM . Recurrent or persistent hyperparathyroidism. J Bone Miner Res. (2002) 17:158–62. 12412795

[5] Udelsman R . Approach to the patient with persistent or recurrent primary hyperparathyroidism. J Clin Endocrinol Metab. (2011) 96:2950–58. doi: 10.1210/jc.2011-1010 21976743

[6] Sahli ZT Karipineni F Zeiger MA . A garden of parathyroid adenomas. BMJ. (2017) 3:1–5. doi: 10.1136/bcr-2017-221130 PMC5747797 28775107

[7] Noussios G Anagnostis P Natsis K . Ectopic parathyroid glands and their anatomical, clinical and surgical implications. Exp Clin Endocrinol Diabetes. (2012) 120:604–10. doi: 10.1055/s-0032-1327628 23174995

[8] Lumachi F Zucchetta P Varotto S Polistina F Favia G D’Amico D . Noninvasive localization procedures in ectopic hyperfunctioning parathyroid tumors. Endocr Relat Cancer. (1999) 6:123–25. doi: 10.1677/erc.0.0060123 10732795

[9] Phitayakorn R McHenry CR . Incidence and location of ectopic abnormal parathyroid glands. Am J Surg. (2006) 191:418–23. doi: 10.1016/j.amjsurg.2005.10.049 16490559

[10] Mendoza V Ramírez C Espinoza AE González GA Peña JF Ramírez ME . Characteristics of ectopic parathyroid glands in 145 cases of primary hyperparathyroidism. Endocr Pract. (2010) 16:977–81. doi: 10.4158/EP10052.OR 20497936

[11] Shen W Düren M Morita E Higgins C Duh QY Siperstein AE . Reoperation for persistent or recurrent primary hyperparathyroidism. Arch Surg. (1996) 131:861–69. doi: 10.1001/archsurg.1996.01430200071013 8712911

[12] Chan TJ Libutti SK McCart JA Chen C Khan A Skarulis MK . Persistent primary hyperparathyroidism caused by adenomas identified in pharyngeal or adjacent structures. World J Surg. (2003) 27:675–79. doi: 10.1007/s00268-003-6812-3 12734681

[13] Stojadinovic A Shriver CD Casler JD Gaertner EM York G Jaques DP . Endoscopic laser excision of ectopic pyriform sinus parathyroid adenoma. Arch Surg. (1998) 133:101–3. doi: 10.1001/archsurg.133.1.101 9438768

[14] Fukumoto A Nonaka M Kamio T Kamura E Ozu C Baba S . A case of ectopic parathyroid gland hyperplasia in the pyriform sinus. Arch Otolaryngol Head Neck Surg. (2002) 128:71–4. doi: 10.1001/archotol.128.1.71 11784259

[15] Dedivitis RA Guimarães AV Pontes GB . Multiple ectopic parathyroid adenomas. Sao Paulo Med J. (2004) 22:32–4. doi: 10.1590/s1516-31802004000100008 PMC11115349 15160525

[16] Murakami N Takeshita A Suzuki H Iizuka T Kikuchi D Matsui A . Hidden culprit of primary hyperparathyroidism. J Clin Endocrinol Metab. (2012) 97:3410–11. doi: 10.1210/jc.2012-2190 22767637

[17] Guevara N Agopian B Benisvy D Lassalle S Santini J Castillo L . Ectopic pyriform sinus parathyroid adenoma. Eur Ann Otorhinolaryngol Head Neck Dis. (2013) 130:95–8. doi: 10.1016/j.anorl.2012.04.008 23021000

[18] Smith MM Young WG Carlin AM . Ghanem TA.Trans-oral robotic surgical excision of an ectopic parathyroid adenoma. J Robot Surg. (2016) 10:73–5. doi: 10.1007/s11701-015-0545-9 26566887

[19] Kim J Cubangbang M Adkins L Chia S DeKlotz TR Boyle L . Ectopic parathyroid adenoma in the pyriform sinus. Head Neck. (2017) 10:110–13. doi: 10.1002/hed.24878 28741786

[20] Muelleman T Yalamanchali S Shnayder Y . Bilateral pyriform sinus parathyroid adenomas. Ear Nose Throat J. (2018) 97:38–40. 29554410

[21] Connolly MJ Lazinski D Aoki KA McLean L Torres C Dos Santos MP . Ectopic parathyroid adenoma in piriform sinus: case report and review of the literature. Ear Nose Throat J. (2019) 98:14–7. doi: 10.1177/0145561318822933 30834784

[22] Hsieh MP Nemer JS Beylergil V Yeh R . Ectopic parathyroid adenoma of the piriform sinus on parathyroid 4D-CT and 99mTc-MIBI SPECT/CT. Clin Nucl Med. (2020) 8:358–59. doi: 10.1097/RLU.0000000000003163 32558723

[23] Zenno A Ramamoorthy B Hammoud DA Quezado M Zeiger MA Jha S . Case Report: Nine-year-old with parathyroid adenoma within the piriform sinus. Front Endocrinol (Lausanne). (2023) 23:1171052. doi: 10.3389/fendo.2023.1171052 PMC10242159 37288292

[24] Sina EM Han CJ Cottrill EE . Undescended superior parathyroid: A case report. Clin Case Rep. (2023) 11:1–4. doi: 10.1002/ccr3.7987 PMC10565088 37830070

[25] Joseph MP Nadol JB Pilch BZ Goodman ML . Ectopic parathyroid tissue in the hypopharyngeal mucosa (pyriform sinus). Head Neck Surg. (1982) 5:70–4. doi: 10.1002/hed.2890050112 7174345

[26] Petranović Ovčariček P Giovanella L Carrió Gasset I Hindié E Huellner MW Luster M . The EANM practice guidelines for parathyroid imaging. Eur J Nucl Med Mol Imaging. (2021) 48:2801–22. doi: 10.1007/s00259-021-05334-y PMC8263421 33839893

[27] Patel DD Bhattacharjee S Pandey AK Kopp CR Ashwathanarayana AG Patel HV . Comparison of 4D computed tomography and F-18 fluorocholine PET for localisation of parathyroid lesions in primary hyperparathyroidism: A systematic review and meta-analysis. Clin Endocrinol. (2023) 99:262–71. doi: 10.1111/cen.14875 36593125

[28] Weinstein GS O’malley BW Jr Hockstein NG . Transoral robotic surgery: supraglottic laryngectomy in a canine model. Laryngoscope. (2005) 115:1315–19. doi: 10.1097/01.MLG.0000170848.76045.47 15995528

